# Outcomes of Liver Transplant Versus Partial Hepatectomy for Perihilar Cholangiocarcinoma Patients Requiring Arterial Reconstruction

**DOI:** 10.1111/liv.70729

**Published:** 2026-06-21

**Authors:** Edoardo Poletto, Pim B. Olthof, Andrea Ruzzenente, Jean‐Philippe Adam, Ian P. J. Alwayn, Emmanuel Boleslawski, Marieke T. de Boer, Alexandre Chebaro, Laurence Chiche, Cristina Dopazo, Jeroen Dubbeld, Joris I. Erdmann, Abdul Hakeem, Minneke J. Coenraad, Hendrien Kuipers, Jean‐Yves Mabrut, Charlotte Maulat, Shishir K. Maithel, Vincent E. de Meijer, Kayvan Mohkam, Sarwa Darwish Murad, Fabrice Muscari, Jens Rolinger, Wojciech G. Polak, Michel Rayar, Francesca Ratti, Fabien Robin, Andreas A. Schnitzbauer, Ernesto Sparrelid, Frederike G. I. Van Vilsteren, Robert J. Porte, Frederik J. H. Hoogwater, Bas Groot Koerkamp, the Perihilar Cholangiocarcinoma Collaboration Group, L. Aldrighetti, W. O. Bechstein, M. A. de Boer, S. Büttner, I. Capobianco, R. Charco, L. C. Franken, S. Gilg, C. Gomez‐Gavara, A. Guglielmi, T. M. van Gulik, J. Heil, H. Jansson, G. Kazemier, P. Lodge, R. Marino, S. Nadalin, T. A. Nguyen, R. Prasad

**Affiliations:** ^1^ Department of Surgery, Dentistry, Gynaecology and Paediatrics, Division of General and Hepato‐Biliary Surgery University of Verona Verona Italy; ^2^ Department of Surgery, Division of Hepato‐Pancreato‐Biliary and Transplant Surgery Erasmus MC Transplant Institute University Medical Center Rotterdam Groningen the Netherlands; ^3^ Department of Surgery, Division of Hepato‐Pancreato‐Biliary Surgery and Liver Transplantation University of Groningen, University Medical Center Groningen Groningen the Netherlands; ^4^ Department of Hepatobiliary and Pancreatic Surgery and Transplantation University Hopital Bordeaux Bordeaux France; ^5^ Department of Surgery, Division of Transplantation Surgery, LUMC Transplant Center Leiden University Medical Center Leiden the Netherlands; ^6^ Department of Digestive Surgery and Transplantation, CHU Lille University of Lille Lille France; ^7^ Department of HBP Surgery and Transplants, Vall D'hebron Hospital Universitari, Vall D'hebron Institut de Recerca (VHIR), Vall D'hebron Barcelona Hospital Campus Universitat Autónoma de Barcelona Barcelona Spain; ^8^ Department of Surgery, Cancer Center Amsterdam Amsterdam UMC, University of Amsterdam Amsterdam the Netherlands; ^9^ Division of Surgery, Department of Hepatobiliary and Liver Transplant Surgery St James's University Hospital Leeds UK; ^10^ Department of Gastroenterology and Hepatology, LUMC Transplant Center Leiden University Medical Center Leiden the Netherlands; ^11^ Department of Digestive Surgery and Liver Transplantation Hôpital De La Croix Rousse Lyon France; ^12^ Department of Digestive Surgery and Liver Transplantation Toulouse‐Rangueil University Hospital Toulouse France; ^13^ Division of Surgical Oncology, Winship Cancer Institute Emory University Atlanta Georgia USA; ^14^ Department of Gastroenterology and Hepatology, Erasmus MC Transplant Institute University Medical Center Rotterdam Rotterdam the Netherlands; ^15^ Department of General, Visceral and Transplant Surgery University HospitalTübingen Tübingen Germany; ^16^ Department of Hepatology Rennes University Hospital Rennes France; ^17^ Institut national de la Santé et de la Recherche Médicale (Inserm) Rennes France; ^18^ Hepatobiliary Surgery Division IRCCS San Raffaele Hospital Milan Italy; ^19^ Service de Chirurgie Hépatobiliaire et Digestif Centre Hospitalier Universitaire de Rennes Rennes France; ^20^ Universitätsklinikum Frankfurt Klinik für Allgemein‐, Viszeral‐ Und Transplantationschirurgie Frankfurt am Main Germany; ^21^ Division of Surgery and Oncology, Department of Clinical Science, Intervention and Technology, Karolinska Institute Karolinska University Hospital Stockholm Sweden; ^22^ Department of Gastroenterology, Division of Hepatology, University of Groningen University Medical Center Groningen Groningen the Netherlands

**Keywords:** biliary tract tumours, cholangiocarcinoma, liver resection, transplantation surgery, vascular reconstruction, vascular resection

## Abstract

**Background and Aims:**

Liver resection (LR) and orthotopic liver transplantation (OLT) are therapeutic options for locally advanced perihilar cholangiocarcinoma (pCCA) requiring hepatic artery reconstruction (HAR). This study aimed to compare short‐ and long‐term outcomes of LR and OLT. Outcomes were major vascular complications, 90‐day mortality, overall survival (OS) and recurrence‐free survival (RFS).

**Methods:**

A cohort of patients undergoing LR with HAR from 10 Western centres was compared with an OLT cohort comprising patients who received or did not receive neoadjuvant chemoradiotherapy (NACR).

**Results:**

109 patients, 60 LR and 49 OLT (22 OLT no‐NACR and 27 OLT NACR) were included. LR patients were older and had fewer Bismuth type 4 tumours (38.3% vs. 69.4%, *p* = 0.009). Positive margins (49.2% vs. 6.5%, *p* < 0.001) and lymph nodes (54.2% vs. 32.4%, *p* = 0.058) were found more frequently in LR patients. No differences were found between LR and OLT in major (40% vs. 46.9%, *p* = 0.56) and vascular complications (23.3% vs. 28.6%, *p* = 0.66); NACR was an independent prognostic factor for vascular complications (OR 2.63, 95% CI 1.03–6.70, *p* = 0.043). 90‐day mortality (15% for LR vs. 10.2% for OLT, *p* = 0.57) and 5‐year OS (HR 0.68, 95% CI 0.40–1.17, *p* = 0.17) were similar. Median OS after LR versus OLT was higher but not significant (24 vs. 40 months, *p* = 0.13). OLT had better 5‐year RFS (HR 0.52, 95% CI 0.29–0.96, *p* = 0.035) than LR. R1 resection (HR 2.07, 95% CI 1.03–4.18, *p* = 0.041) and perineural invasion (HR 3.64, 95% CI 1.09–12.16, *p* = 0.035) were independent prognostic factors for RFS.

**Conclusions:**

LR and OLT for locally advanced pCCA had similar rates of major complications and post‐operative mortality, but NACR was associated with increased vascular complications. Survival was difficult to compare in the groups due to their heterogeneity, but OLT, especially with NACR, seems to give better results than LR.

## Lay Summary

- LR with HA reconstruction and OLT are both valuable options for locally advanced pCCA.
- There was no difference in postoperative mortality and morbidity found between LR and OLT.
- Neo‐adjuvant chemo‐radiotherapy was associated with increased morbidity
- LR with HA reconstruction and OLT are associated with high morbidity and mortality, the correct treatment must be allocated to the patients after careful consideration of tumour biology (i.e., nodal involvement).

**Abbreviations**
HAR: hepatic artery reconstruction
LR: liver resection
NACR: neoadjuvant chemo‐radiotherapy
OLT: orthotopic liver transplantation
pCCA: perihilar cholangiocarcinoma
PHLF: post‐hepatectomy liver failure
PSC: primary sclerosing cholangitis
PVR: portal vein reconstruction
VR: vascular resection and reconstruction

## 1 Introduction

If technically possible, complete resection remains the best therapeutic option for patients with perihilar cholangiocarcinoma (pCCA), with a 5‐year overall survival (OS) between 30% and 50% [1, 2, 3, 4]. Only a minority of patients (20%) are eligible for surgery [5, 6, 7], because most patients have metastatic or locally advanced disease at diagnosis or are too frail to undergo major surgery.

One of the main reasons for defining a pCCA as locally advanced is the involvement of the portal vein or hepatic artery in the future liver remnants. Vascular resection and reconstruction (VR) during liver resection (LR) can increase the number of patients eligible for surgery; however, its use is still debated [8, 9]. Hepatic artery reconstruction (HAR) has been associated with a large increase in postoperative morbidity and mortality in several studies [8, 10]. Other studies, however, found that the benefit of LR in OS justified the increased risk of complications after HAR [11, 12, 13].

Orthotopic liver transplantation (OLT) has been proposed to achieve the most radical resection of locally advanced pCCA. The Mayo Clinic protocol involves neoadjuvant chemoradiation (NACR) [14]. This protocol applies strict patient selection criteria that are met by only about 4% of all patients with pCCA [14, 15]. In a retrospective cohort of 237 patients transplanted for pCCA from 1992 to 2019, Azad et al. reported a 5 year intention‐to‐treat OS of 68%; in their cohort 84 (35%) patients had de novo pCCA, 153 (65%) had PSC‐associated pCCA and OS was significantly longer for the second group (58% vs. 74%) [16]. NACR, especially the radiation component, has been associated with a high risk of vascular complications such as hepatic artery thrombosis or haemorrhage, with rates as high as 23% [17, 18]. A comparison of VR outcomes between patients with locally advanced pCCA undergoing LR and OLT preceded or not preceded by NACR is lacking in the literature and has been previously advocated [11].

This study aimed to compare LR and OLT (with or without NACR) for patients with locally advanced pCCA in terms of major and vascular complications, 90‐day mortality, OS, and recurrence‐free survival (RFS) in patients with locally advanced pCCA.

## 2 Patients and Methods

### 2.1 Study Design

This observational cohort study included retrospectively collected data from two separate collaborative study groups. Data from patients undergoing surgical exploration at 25 European and American centres for proven or suspected pCCA during any time span not preceding the year 2000 were included in a collaborative retrospective, standardized, and de‐identified database of the Perihilar Cholangiocarcinoma Collaboration Group. The participating centres in this study provided additional information on VR and vascular complications. Only patients with histologically proven pCCA in surgical specimens were included. HAR was the focus of the comparison given that this is a reconstruction with a higher risk of complications. Therefore, only patients who underwent HAR with or without associated portal vein reconstruction (PVR) were included. Patients undergoing bile duct resection without partial hepatectomy, those not undergoing VR, and those undergoing PVR only were excluded [19].

Data for the OLT cohort were obtained from an international, multicentre, retrospective database of consecutive patients who underwent OLT for locally advanced pCCA unsuitable for LR between April 2011 and July 2020 [20]. Patients were selected for OLT based on strict Mayo Clinic selection criteria [21]. The population of the aforementioned study was divided into a Dutch cohort of patients who did not receive NACR (OLT no‐NACR group), and a French cohort of patients who received NACR (OLT‐NACR group), and a subgroup analysis comparing LR with OLT with or without NACR was conducted. According to the Central Committee on Research Involving Human Subjects, no written consent has been obtained from the patients as there is no patient‐identifiable data included.

The endpoints of this study were major and vascular complications, 90‐day mortality, OS, and RFS.

### 2.2 Definitions

The work‐up and management of patients, as well as postoperative management and follow‐up, differed across centres and during the inclusion period, according to the protocols of each institution. The criteria for defining unrespectability, diagnostic criteria, and selection criteria in the OLT cohort have been previously described [20]. Patients in the LR cohort were defined as locally advanced because of the requirement for hepatic artery reconstruction of the remnant liver. Involvement could pertain to the main hepatic artery, as well as the unilateral hepatic artery to the remnant liver.

Tumours were classified according to the Bismuth‐Corlette classification [22]. Liver resections were defined according to the Brisbane terminology [23]; negative resection margins were defined as tumour‐free margins in all resection planes in the pathology report. HAR was defined as any resection of the left, right, or main hepatic artery to the future liver remnant followed by reconstruction due to vascular involvement. All complications registered during initial hospitalization or within 90 days after surgery were reported and classified according to the Clavien‐Dindo classification [24]. Vascular complications were recorded for both LR and OLT, and included thrombosis, post‐operative haemorrhage, pseudoaneurysm, and stenosis. Major vascular complications were defined as Clavien–Dindo grade > 3a. The definitions and grading for post‐hepatectomy liver failure (PHLF) and bile leak proposed by the International Study Group of Liver Surgery (ISGLS) were used [25, 26]. Only grades B and C were considered clinically relevant. Other biliary complications analysed included biliary stenosis, necrosis, and cholangitis. OS was defined as the time between surgery and death or the last follow‐up, whereas RFS was defined as the time between surgery and the first radiological or pathological evidence of recurrence.

### 2.3 Statistical Analysis

Categorical data were presented as frequencies and percentages and compared using Pearson's χ squared test or Fisher's exact test, when appropriate. Continuous variables are presented as median and interquartile range (IQR) and compared using the Mann–Whitney U test. Logistic regression was used to identify independent risk factors for vascular complications. OS and RFS were analysed using Kaplan–Meier curves and compared between the groups using the log‐rank test. Cox regression analysis was performed to investigate the prognostic factors for RFS and OS. Factors with a *p* < 0.20 at univariate analysis were included in multivariate analysis with backwards stepwise selection, both for logistic and Cox regression analysis. Patients from the transplant cohort with no malignancy in the resected liver and no malignancy in the pre‐transplant histological examination were excluded from the survival analyses. For all analyses, two‐sided *p*‐values < 0.05 were considered significant. All analyses were performed, and all figures were produced using SPSS (software version 28, IBM, US).

## 3 Results

**FIGURE 1:**
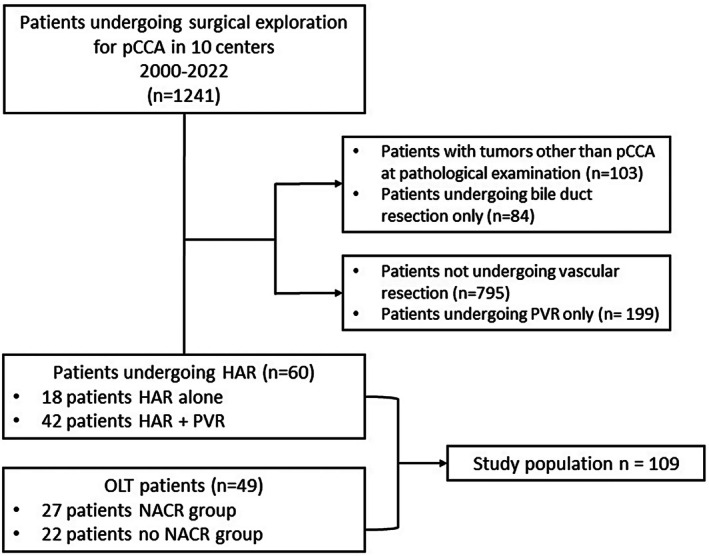
Flowchart showing the selection of the study population.

A total of 10 out of 25 centres provided the additional data required for this study, representing 1241 patients undergoing LR for pCCA. Subsequent exclusion was required for 103 patients (8.3%) who had a final pathological diagnosis other than pCCA and 84 patients (6.8%) who underwent bile duct resection without partial hepatectomy. Of the remaining 1054 patients, 795 (75.4%) did not undergo VR and 199 (18.9%) underwent PVR only. The final LR cohort consisted of 60 patients with locally advanced pCCA who underwent HAR, including 42 (70%) who underwent combined PVR and HAR (Figure 1). The OLT cohort consisted of 49 patients: 27 (55.1%) in the NACR group and 22 (44.9%) in the no‐NACR group. The study population consisted of 109 patients.

### 3.1 Patients, Operative and Pathological Characteristics

Patients undergoing LR were older than those undergoing OLT (66 vs. 54, *p* < 0.001) and suffered less from Primary Sclerosing Cholangitis (PSC) (1.7% vs. 28.6%, *p* < 0.001) (Table 1). Tumours in the LR group were less frequently Bismuth Type 4 than in the OLT group (38.3% vs. 69.4%, *p* = 0.009), but this was less evident when comparing LR with OLT no‐NACR (63.6%, *p* = 0.24). Additional pancreatoduodenectomy was performed in none of the patients (0%) in the LR group and in 4 (8.2%) patients in the OLT group (*p* = 0.027). No differences were found in median tumour diameter in the pathological report (2.9 vs. 2.6 cm, *p* = 0.94) or in the proportion of patients with tumours larger than 3 cm (32.7% for LR and 28.6% for OLT, *p* = 0.65). Positive lymph nodes were reported in 54.2% of the LR patients and 32.4% of the OLT patients (*p* = 0.058). Finally, a positive margin (R1 resection) was more frequent in LR patients than in OLT patients (49.2% vs. 6.5%, *p* < 0.001), for both OLT no NACR (10.5%, *p* = 0.003) and NACR (3.7%, *p* < 0.001) patients.

**TABLE 1:** Demographic, operative and pathological characteristics of the study population, divided into three groups: Liver resection (LR), orthotopic liver transplantation (OLT) not preceded by neo chemoradiotherapy (NACR) and preceded by NACR (OLT no NACR and OLT NACR respectively).

Variable	LR (*n* = 60)	OLT (*n* = 49)	LR vs. OLT	OLT no NACR (*n* = 22)	OLT NACR (*n* = 27)	LR vs. OLT no NACR	LR vs. OLT NACR
Age	66 (59–73)	54 (46–60)	*< 0.001* ^*a*^	56 (39–61)	53 (48–60)	*< 0.001* ^*a*^	*< 0.001* ^*a*^
Sex, male	41 (68.3%)	34 (69.4%)	0.99	14 (63.6%)	20 (74.1%)	0.79	0.80
BMI	26 (22–28)	24 (21–25)	*0.021* ^*a*^	23 (21–25)	24 (22–25)	*0.039* ^*a*^	0.080^a^
CA 19–9 (UI/mL)	125 (23–397)	143 (13–833)	0.80^a^	94 (8–717)	159 (52–1331)	0.78^a^	0.41^a^
PSC	1 (1.7%)	14 (28.6%)	*< 0.001*	11 (50.0%)	3 (11.1%)	*< 0.001* ^*b*^	0.052
Preoperative biliary drainage							
None	9 (15.0%)	4 (8.2%)		1 (4.5%)	3 (11.1%)		
PBD	22 (36.7%)	13 (26.5%)		0 (0%)	13 (48.1%)		
EBD	25 (41.7%)	32 (65.3%)		21 (35.5%)	11 (40.7%)		
Both	4 (6.7%)	0 (0%)	*0.045* ^*b*^	0 (0%)	0 (0%)	*< 0.001* ^*b*^	0.45^b^
Bismuth type							
I‐II	7 (11.7%)	0 (0%)		0 (0%)	0 (0%)		
IIIa	11 (18.3%)	7 (14.3%)		3 (13.6%)	4 (14.8%)		
IIIb	19 (31.7%)	8 (16.3%)		5 (22.7%)	3 (11.1%)		
IV	23 (38.3%)	34 (69.4%)	*0.009* ^*b*^	14 (63.6%)	20 (74.1%)	0.24^b^	*0.024* ^*b*^
PVE	13 (21.7%)	—	—	—	—	—	—
Resection type							
Left Hemihep.	25 (41.7%)	—					
Right Hemihep.	19 (31.7%)	—					
Left Extended Right Extended	7 (11.6%) 6 (10.0%)	— —					
Central	3 (5.0%)	—					
Transplantation		49 (100%)	—	22 (100%)	27 (100%)	—	—
Pancreatoduodenectomy	0 (0%)	4 (8.2%)	*0.027* ^*b*^	3 (13.6%)	1 (3.7%)	*0.004* ^*b*^	0.14^b^
Type of VR							
HAR only	18 (30%)						
PVR + HAR	42 (70%)		—	22 (100%)	27 (100%)	—	—
Tumour diameter (cm)	2.9 (2.0–3.5)	2.6 (1.8–4.3)	0.94^a^	2.6 (1.8–3.5)	2.5 (2.0–5.0)	0.66^a^	0.64^a^
Diameter ≥ 3 cm	17 (32.7%)	14 (28.6%)	0.65	5 (22.7%)	9 (33.3%)	0.96^a^	0.43
R1 resection margin	29 (49.2%)	3 (6.5%)	*< 0.001*	2 (10.5%)	1 (3.7%)	*0.003*	*< 0.001*
Positive Lymph nodes	32 (54.2%)	12 (32.4%)	0.058	7 (36.8%)	5 (27.8%)	0.29	0.060
Perineural invasion	56 (94.9%)	33 (71.7%)	*0.002*	11 (57.9%)	22 (81.5%)	*< 0.001*	*0.047* ^*b*^

*Note:* Categorical variables are expressed as frequencies and percentages, and continuous variables are expressed as median and interquartile range (IQR). The *p*‐value refers to Fisher's exact test when not specified. TTS: time to surgery, time from surgical exploration to OLT. Different percentages may be related to the same value frequencies owing to missing values.

Abbreviations: BMI, body mass index; HAR, hepatic artery reconstruction; EBD, endoscopic biliary drainage; PBD, percutaneous biliary drainage; PSC, primary sclerosing; PVE, portal vein embolization, PVR, portal vein reconstruction.

^a^ Mann Whitney *U* test.

^b^ Pearson's Chi squared test.

### 3.2 Postoperative Complications

No differences were found between LR and OLT (with or without NACR) in major (40% vs. 46.9%, *p* = 0.56) or vascular complications (23.0% vs. 28.6%, *p* = 0.66) (Table 2). Postoperative mortality within 90 days was also comparable between the two groups (15% vs. 10.2%, *p* = 0.57). PHLF was more frequent in LR patients than in OLT patients (20% vs. 0%, *p* < 0.001), whereas a difference in severe biliary complications could not be demonstrated against patients undergoing OLT with NACR (16.7% vs. 25.9%, *p* = 0.38); on the other hand, patients undergoing OLT with no NACR had a higher incidence of biliary complications (16.7% vs. 40.9%, *p* = 0.036). However, severe biliary complications were less common when comparing LR to OLT no‐NACR (16.7% vs. 40.9%, *p* = 0.036). NACR was the only independent prognostic factor for the occurrence of vascular complications in the study population (OR 2.63, 95% CI 1.03–6.70, *p* = 0.043; Table 3). NACR patients had more frequent vascular complications (40.7% vs. 20.7%, *p* = 0.046) and hepatic artery complications (33.3% vs. 14.6%, *p* = 0.048) than non‐NACR patients (Table 4).

**TABLE 2:** Postoperative course of the study population, divided into three groups: Liver resection (LR), orthotopic liver transplantation (OLT) not preceded by neo adjuvant chemo‐radiotherapy (NACR) and preceded by NACR (OLT no NACR and OLT NACR respectively).

Variable	LR (*n* = 60)	OLT (*n* = 49)	LR vs. OLT	OLT no NACR (*n* = 22)	OLT NACR (*n* = 27)	LR vs. OLT no NACR	LR vs. OLT NACR
Length of stay, days	16 (11–28)	14 (10–20)	0.13^a^	13 (10–15)	15 (10–22)	0.14^a^	0.31^a^
Major complications (CD ≥ 3)	24 (40.0%)	23 (46.9%)	0.56	11 (50%)	12 (44.4%)	0.46	0.81
PHLF grade B/C (ISGLS)	12 (20%)	0 (0%)	*< 0.001*	0 (0%)	0 (0%)	*0.023* ^b^	*0.012* ^b^
Bile leak grade B/C (ISGLS)	10 (16.7%)	9 (18.4%)	0.99	4 (18.2%)	5 (18.5%)	0.87^b^	0.83^b^
Severe biliary complications	10 (16.7%)	16 (32.7%)	0.071	9 (40.9%)	7 (25.9%)	*0.036*	0.38
Stenosis	0 (0%)	7 (14.3%)		5 (22.7%)	2 (7.4%)		
Leakage	10 (16.7%)	9 (18.4%)		4 (18.2%)	5 (18.5%)		
Necrosis	0 (0%)	1 (2.0%)		0 (0%)	1 (3.7%)		
Cholangitis	0 (0%)	2 (4.1%)		1 (4.5%)	1 (3.7%)		
Severe Infectious Complications	10 (16.7%)	10 (20.4%)	0.62	6 (27.3%)	4 (14.7%)	0.83	0.35
Vascular Complications	14 (23.3%)	14 (28.6%)	0.66	3 (13.6%)	11 (40.7%)	0.34^b^	0.13
Portal Vein Complication^c^	4 (9.5%)	7 (14.3%)	0.54	2 (9.1%)	5 (18.5%)	0.95^b^	0.28^b^
Stenosis	2 (4.7%)	3 (6.1%)		0 (0%)	3 (11.1%)		
Thrombosis	2 (4.7%)	4 (8.2%)		2 (9.1%)	2 (7.4%)		
Hepatic Artery Complications	10 (16.7%)	11 (22.4%)	0.47	2 (9.1%)	9 (33.3%)	0.39^b^	0.098
Bleeding	2 (3.3%)	6 (12.2%)		2 (9.1%)	4 (14.8%)		
Stenosis	1 (1.7%)	2 (4.1%)		1 (4.5%)	1 (3.7%)		
Aneurysm	2 (3.3%)	3 (6.1%)		0 (0%)	3 (11.1%)		
Thrombosis	6 (10.0%)	1 (2.0%)		0 (0%)	1 (3.7%)		
90 Days mortality, cause.	9 (15%)	5 (10.2%)	0.57	1 (4.5%)	4 (14.8%)	0.20^b^	0.98^b^
PHLF	2 (3.3%)	0 (0.0%)		0 (0%)	0 (0%)		
Sepsis	3 (5.0%)	0 (0.0%)		0 (0%)	0 (0%)		
MOF	1 (1.7%)	2 (4.1%)		1 (4.5%)	1 (3.7%)		
Bleeding	3 (5.0%)	3 (6.1%)		0 (0%)	3 (11.1%)		

*Note:* Categorical variables are expressed as frequencies and percentages, and continuous variables are expressed as median and interquartile range (IQR). The *p*‐value refers to Fisher's exact test when not specified. Frequencies of single complications may not add up to the reported total, given that a patient may have more than one complication per type.

Abbreviations: CD, Clavien‐Dindo; MOF, multi organ failure; PHLF, post‐hepatectomy liver failure.

^a^ Mann Whitney *U* test.

^b^ Pearson's Chi squared test.

^c^ Portal Vein complications were evaluated only in patients with LR undergoing both PVR and HAR.

**TABLE 3:** Logistic regression analysis for vascular complications.

Variable	Univariate		Multivariable	
	OR (95% CI)	*p*	OR (95% CI)	*p*
Age	0.99 (0.97–1.01)	0.20	—	—
Sex, male	1.32 (0.83–2.09)	0.24	—	—
BMI	1.10 (0.97–1.24)	0.13	1.13 (0.99–1.28)	0.07
CA 19–9 (UI/mL)	1.00 (1.00–1.00)	0.97	—	—
PSC	0.69 (0.18–2.65)	0.60	—	—
Preoperative biliary drainage	0.50 (0.15–1.69)	0.27	—	—
Preoperative cholangitis	0.53 (0.14–1.94)	0.35	—	—
Bismuth Type IV	1.30 (0.55–3.09)	0.55	—	—
NACR	2.63 (1.03–6.70)	0.043	2.63 (1.03–6.70)	0.043
OLT (vs. LR)	1.31 (0.55–3.11)	0.53	—	—

Abbreviations: BMI, body mass index; LR, liver resection; NACR, neo adjuvant chemoradiotherapy; OLT, orthotopic liver transplantation; PSC, primary sclerosing cholangitis.

**TABLE 4:** Postoperative course of the study population divided based on preoperative neo adjuvant chemo‐radiotherapy (NACR).

Variable	No NACR (*n* = 82)	NACR (*n* = 27)	*p*
Length of stay, days	15 (10–26)	15 (10–22)	0.56^a^
Major complications (CD ≥ 3)	35 (42.7%)	12 (44.4%)	1.00
PHLF grade B/C (ISGLS)	12 (14.6%)	0 (0.0%)	*0.035* ^b^
Severe biliary complications	19 (23.2%)	7 (25.9%)	0.80
Stenosis	5 (6.1%)	2 (7.4%)	
Leakage	14 (17.1%)	5 (18.5%)	
Necrosis	0 (0%)	1 (3.7%)	
Cholangitis	1 (1.2%)	1 (3.7%)	
Bile leak grade B/C (ISGLS)	14 (17.1%)	5 (18.5%)	0.86^b^
Vascular complications	17 (20.7%)	11 (40.7%)	*0.046*
Portal vein complication^c^	6 (9.4%)	5 (18.5%)	0.22^b^
Stenosis	2 (3.1%)	3 (11.1%)	
Thrombosis	4 (6.3%)	2 (7.4%)	
Hepatic artery complications	12 (14.6%)	9 (33.3%)	*0.048*
Bleeding	4 (4.9%)	4 (14.8%)	
Stenosis	2 (2.4%)	1 (3.7%)	
Aneurysm	2 (2.4%)	3 (11.1%)	
Thrombosis	6 (7.3%)	1 (3.7%)	
90 Days mortality	10 (12.2%)	4 (14.8%)	0.72^b^
PHLF	2 (2.4%)	0 (0%)	
Sepsis	3 (3.7%)	0 (0%)	
MOF	2 (2.4%)	1 (3.7%)	
Bleeding	3 (3.7%)	3 (11.1%)	

*Note:* Categorical variables are expressed as frequencies and percentages, and continuous variables are expressed as median and interquartile range (IQR). *p*‐values refer to Fisher's exact test when not specified differently. Frequencies of single complications may not add up to the reported total, given that a patient may have more than one complication per type.

Abbreviations: CD, Clavien Dindo; MOF, multiorgan failure; PHLF, post‐hepatectomy liver failure.

^a^ Mann Whitney *U* test.

^b^ Pearson's Chi squared test.

^c^ Portal Vein complications were evaluated only in patients with LR undergoing both PVR and HAR.

A multivariate analysis for 90‐day mortality was also conducted: age (OR 1.08, 95% CI 1.01–1.17, *p* = 0.027) and vascular complications (OR 9.60, 95% CI 2.16–42.60, *p* = 0.003) were independent risk factors, while preoperative biliary drainage was an independent protective factor (OR 0.15, 95% CI 0.03–0.73, *p* = 0.019) (Table S1).

### 3.3 Survival Analysis

**FIGURE 2:**
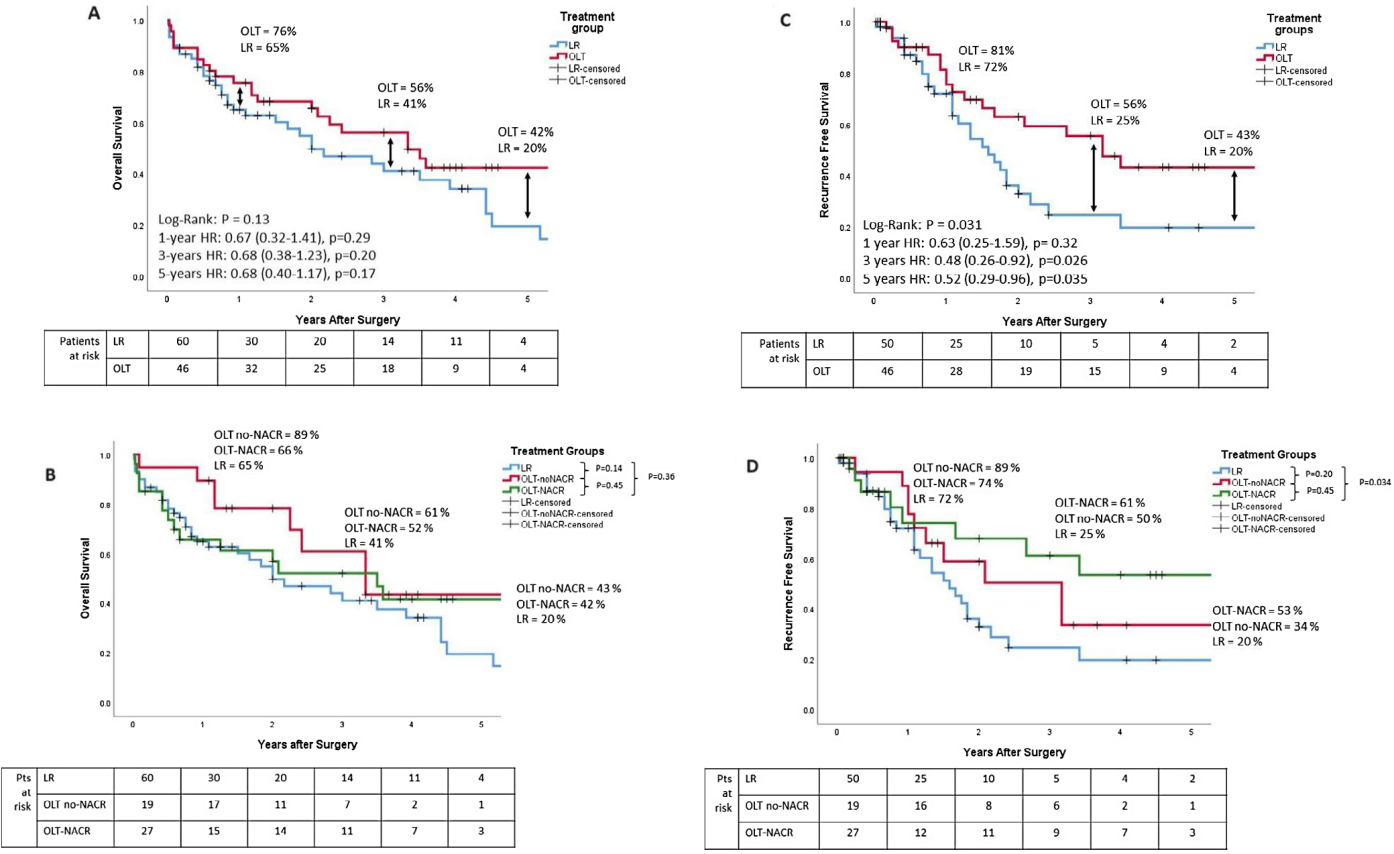
Overall survival of the study population. (A) LR vs. OLT (B) LR vs. OLT no NACR vs. OLT NACR. Recurrence free survival, (C) LR vs. OLT (D) LR vs. OLT no NACR vs. OLT NACR. In Figure 2B the Log rank test for OS never gave a statistically significant result (LR vs. OLT no NACR *p* = 0.14, LR vs. OLT NACR *p* = 0.36, OLT no NACR vs. NACR p = 0.45), while in Figure 2D the test for RFS gave a significant difference for LR compared to OLT with NACR (*p* = 0.034), but not for the other comparisons (LR vs. OLT no NACR *p* = 0.20, OLT NACR vs. OLT no NACR *p* = 0.45).

No difference in the median OS after LR versus OLT was observed (24 vs. 40 months, *p* = 0.13). OS at 3‐year was 41% for LR versus 56% for OLT (log‐rank *p* = 0.20; Figure 2A). No differences were found when comparing LR with OLT no NACR (3‐ and 5‐years OS 61% and 43%, respectively; *p* = 0.14) and LR with OLT NACR (3‐ and 5‐years OS 52% and 42%, respectively; *p* = 0.36; Figure 2B). BMI (HR 2.81, 95% CI 1.13–7.02, *p* = 0.026) and nodal involvement (HR 2.56, 95% CI 1.24–5.31, *p* = 0.011) were independent poor prognostic factors for OS (Table 5). The median RFS after LR was lower than that after OLT (19 vs. 38 months, *p* = 0.031; Figure 2C); OLT NACR showed the best RFS that was significantly higher than that of LR (3‐ and 5‐years RFS 61% vs. 25% and 53% vs. 20%, respectively, *p* = 0.034), but not higher than OLT no NACR RFS (3‐ and 5‐years RFS 50% and 34%, respectively, *p* = 0.45, Figure 2D). Independent poor prognostic factors for RFS included positive resection margin (HR 2.64, 95% CI 1.18–5.91) and perineural invasion (HR 3.64, 95% CI 1.09–12.16, *p* = 0.035) (Table 6). Post recurrence OS did not differ between the two groups (LR 7 months, IQR 3–38 vs. OLT 11 months, IQR 3–14, *p* = 0.53); data on post recurrence management was not available for enough patients to conduct a proper analysis.

**TABLE 5:** Cox regression analysis for overall survival.

Variable	Univariate		Multivariable	
	HR (95% CI)	*p*	HR (95% CI)	*p*
Age	1.02 (0.99–1.04)	0.17	—	—
Sex, male	0.98 (0.55–1.75)	0.95	—	—
BMI ≥ 30	2.45 (1.13–5.33)	0.023	2.81 (1.13–7.02)	0.026
CA 19–9 (UI/mL)	1.00 (1.00–1.00)	0.45	—	—
PSC	0.68 (0.30–1.59)	0.37	—	—
Preoperative biliary drainage	0.75 (0.35–1.60)	0.46	—	—
Bismuth Type IV	1.05 (0.62–1.77)	0.86	—	—
Pancreatoduodenectomy	0.59 (0.14–2.43)	0.47	—	—
Vascular complications	1.17 (0.56–2.45)	0.68	—	—
Tumour diameter (cm)	1.13 (0.98–1.30)	0.091	1.18 (0.94–1.49)	0.15
R1 resection margin	1.71 (0.97–3.02)	0.065	0.97 (0.42–2.24)	0.95
Positive Lymph nodes	1.91 (1.08–3.39)	0.027	2.21 (1.13–4.43)	0.025
Perineural invasion	2.81 (1.01–7.77)	0.047	1.74 (0.52–5.89)	0.37
NACR	0.84 (0.46–1.54)	0.57	—	—
OLT (vs. LR)	0.66 (0.39–1.13)	0.13	0.93 (0.44–1.97)	0.85

Abbreviations: BMI, body mass index; LR, liver resection; NACR, neoadjuvant chemoradiotherapy; OLT, orthotopic liver transplantation; PSC, primary sclerosing cholangitis.

**TABLE 6:** Cox regression analysis for recurrence free survival in the study population.

Variable	Univariate		Multivariable	
	HR (95% CI)	*p*	HR (95% CI)	*p*
Age	1.01 (0.99–1.04)	0.33	—	—
Sex, male	0.79 (0.42–1.50)	0.47	—	—
BMI ≥ 30	2.31 (0.89–6.02)	0.086	2.56 (0.96–6.84)	0.06
CA 19–9 (UI/mL)	1.00 (1.00–1.00)	0.48	—	—
PSC	0.67 (0.28–1.85)	0.36	—	—
Preoperative biliary drainage	1.36 (0.49–3.80)	0.57	—	—
Bismuth Type IV	1.14 (0.64–2.04)	0.65	—	—
Pancreatoduodenectomy	0.76 (0.18–3.15)	0.71	—	—
Vascular complications	0.89 (0.40–2.00)	0.79	—	—
Tumour diameter (cm)	1.11 (0.94–1.31)	0.21	—	—
R1 resection margin	2.47 (1.32–4.62)	0.005	2.07 (1.03–4.18)	0.041
Positive Lymph nodes	1.48 (0.81–2.71)	0.21	—	—
Perineural invasion	3.12 (1.12–8.75)	0.030	3.64 (1.09–12.16)	0.035
NACR	0.49 (0.23–1.06)	0.072	0.61 (0.26–1.46)	0.27
OLT (vs. LR)	0.52 (0.29–0.96)	0.035	2.18 (0.84–5.61)	0.11

Abbreviations: BMI: Body mass index; LR: liver resection; NACR: neoadjuvant chemoradiotherapy; OLT: orthotopic liver transplantation; PSC: primary sclerosing cholangitis.

## 4 Discussion

In this multicentre observational cohort study, no difference was observed in postoperative major and vascular complications and 90‐day mortality between patients with locally advanced pCCA undergoing LR with HAR or OLT with or without NACR. PHLF was more common after LR, and severe biliary complications were the most frequent after OLT without NACR. NACR was the only poor prognostic factor for the occurrence of vascular complications (OR, 2.56; 95% CI 1.24–5.31, *p* = 0.011). OLT was associated with a significantly better RFS, although a statistically significant difference in OS could not be demonstrated. BMI ≥ 30 and positive nodal status were independent poor prognostic factors for OS. Positive margins and perineural invasion were independent poor prognostic factors for RFS.

To our knowledge, the present study is the first comparison of LR and OLT, with a focus on vascular complications, in patients with locally advanced pCCA requiring HAR. Several studies have compared the LR for pCCA with and without VR. In their recent single‐centre series of 303 pCCA patients undergoing LR with VR (venous or arterial) vs. 484 patients without VR, Mizuno et al. reported a comparable incidence of severe complications (50% vs. 48%, respectively, *p* = 0.71); in contrast, VR patients showed higher 90‐day mortality (3.6% vs. 1.2%, *p* = 0.04), portal vein complications (4.6% vs. 1%, *p* = 0.011), and hepatic artery complications (8.2% vs. 2%, *p* < 0.001) [11]. Kuriyama et al. found no differences in major complications (37.5% vs. 53.4%, *p* = 0.21) and 90‐day mortality (4.2% vs. 3.4%, *p* = 0.12) between 48 VR patients and 58 non‐VR patients. Both studies showed that in expert centres, VR is feasible and can offer a chance for long‐term OS in patients with locally advanced pCCA [27]. Breuer et al. suggested that OLT should have a lower risk of major complications and postoperative mortality [28].

Our study could not demonstrate a difference between LR and OLT when comparing the risks of major complications, vascular complications, and postoperative mortality within 90 days. The comparable incidence of vascular complications may come as a surprise; however, NACR was a poor independent prognostic factor for vascular complications, with an OR of 2.63. This result is consistent with the available literature that identifies in NACR the probable cause of increased vascular complications after OLT for pCCA [29, 30, 31]. Tan et al. compared 74 patients with OLT and pCCA who received NACR and 173 who underwent transplantation for other causes. They found that vascular complications were more common in OLT for pCCA; in particular, hepatic artery complications increased after OLT for pCCA (24.3% vs. 11.6%, *p* = 0.012) [29].

The incidence of major complications was comparable between the LR and OLT groups (40% vs. 46.9%, *p* = 0.56). This relatively small incidence of major complications, as compared with a benchmark value of ≤ 70% [32], may be due to the fact that among LR patients left hemi‐hepatectomy was the most common type of resection. Left hemi‐hepatectomy is associated with lower morbidity compared to right‐sided resection because of the larger future liver remnant and therefore reduced risk of liver failure [33, 34, 35]. Nonetheless, moderate‐to‐severe PHLF was seen in 20% of the patients, whereas it was completely absent in the transplanted patients. On the other hand, it is interesting to see how biliary complications were significantly more frequent in OLT without NACR compared to LR, while no difference was found when comparing OLT NACR and LR. Although no available study identified NACR as an independent risk or protective factor for biliary complications, it can be speculated that NACR, especially radiotherapy, may “harden” the recipient bile duct, making the anastomosis easier and less prone to reject and mismatch of the duct [36, 37].

Moving onto the pathologist bench, a positive margin was obtained in 49.2% of LR patients and only 6.5% of OLT patients, indicating that OLT may be a more radical therapeutic option for patients with locally advanced pCCA, which may explain the significant difference in RFS reported in our paper (43% for OLT vs. 20% for LR at 5 years, *p* = 0.035), a result that almost equals the benchmark value for RFS set by Breuer et al. in their recent paper (≥ 43.8% at 5 years) [28].

A recent meta‐analysis by Cambridge et al. reported 65.1% 5 years OS for OLT with NACR in pCCA patients [38]. The results reported in our paper are worse (42% 5‐year OS for OLT patients): this can be due to many factors, first of all the high proportion of nodal positive patients in the OLT group (34%, 36.8% in patients transplanted without NACR, 27.8% in patients after NACR); another puzzling result is the lack of positive effect on OS of OLT compared to LR, even after stratifying for NACR: the reason is again multifactorial, starting from the small sample size and many heterogeneities in the distribution of prognostic factors between groups, but again the very similar distribution of N1 between groups surely played a role in it, and this is at the very least a reminder of how “biology is queen” and we should be very careful in allocating patients to the proper treatment based on the better or worse biology of the disease [39]; nevertheless, the curves for OS diverge, especially after the 2nd year, and median OS is quite different (24 vs. 40 months), even without statistical significance, hinting at a possible positive effect of OLT in more controlled conditions. Keeping in mind these limitations, it's also interesting to notice that OLT seems to grant a positive effect on RFS and when stratifying for preoperative treatment the OLT NACR group showed longer RFS than LR (*p* = 0.034), a fact that is not true for OLT alone (*p* = 0.20). This result is difficult to interpret and may hint both at a positive effect on survival given by NACR, but also at a selection of biologically “more favourable” candidates among the OLT candidates; further studies are warranted to better investigate this aspect. Unfortunately, the effect of post recurrence management on OS was impossible to investigate due to the lack of data for most patients, even if post‐recurrence OS did not differ significantly between LR and OLT.

It has been postulated that radiotherapy is responsible for the vascular complications in patients undergoing OLT with NACR [17, 18]. In our cohort NACR was associated with an increased risk of vascular complications that were in turn associated with increased 90‐day mortality. It remains unclear how this side effect is comparable to the potential benefit of radiotherapy using the Mayo protocol, given how our data seem to suggest that NACR potentially reduced the oncological futility but at the cost of indirectly increasing surgical futility. Future studies should consider neoadjuvant regimens that use systemic chemotherapy without RT. This could decrease the risk of vascular complications associated with radiation without sacrificing the potential benefits of a neoadjuvant approach. A definitive claim for superiority or inferiority of OLT on LR, and of NACR or no NACR, however could be made only after a randomized controlled trial: a first attempt was made by the TRANSPHIL trial (clinicaltrials.gov; NCT02232932), that was unfortunately closed prematurely due to failure of recruitment; more recently, a new multicentric randomized study called LITALHICA trial (NCT06125769) was promoted by the university of Padua in Italy [40], and its results are eagerly awaited.

This study had several limitations that must be considered when interpreting the results. First, given the rarity of the disease and the infrequent use of OLT and LR with VR in patients with pCCA, the study population was small, resulting in inadequate power to rule out clinically relevant differences in the outcomes. Second, the LR and OLT groups differed at baseline; because of the limited sample size, we could not control for potential confounders, and because of the retrospective study design, we cannot rule out any unknown remaining confounders; in particular, OLT patients were younger and presented more frequently with pCCA associated with PSC, that is usually associated with better prognosis [38, 41], but, on the other hand, presented more frequently with Bismuth type IV tumours. Moreover, as it was easy to predict, OLT patients achieved a much higher percentage of R0 resection. All these factors must be considered especially in interpreting the results of the survival analysis, that de facto compares two very heterogeneous groups. However, given the circumstances and the limited available literature on this topic, the current results are as good as possible. Third, patients treated with NACR have a pre‐transplant interval in which selection bias occurs because patients with disease progression or clinical deterioration drop out. Finally, the heterogeneity of the two groups made the survival analysis flawed and its results are difficult to interpret. In particular, RFS was longer for OLT NACR patients, but OS was not statistically different, and the lack of post recurrence management data means we cannot evaluate its effect on these results.

All limitations considered, we believe this study confirms how the risk of complications and mortality resides not in the technique used to treat locally advanced pCCA (OLT or LR), but in other factors, of which NACR seems to be the most important. On the other hand, however, NACR seems to have a positive impact on recurrence‐free survival, while the technique of choice had a limited effect given the great negative impact of biological negative prognostic factors like N1. In the end, our findings, provided that patients are treated in high‐volume, highly experienced HPB and liver transplantation centres, urge to carefully select patients that could benefit from OLT, to preferably perform NACR remembering the increased risk of vascular complications, and to always consider radical intent surgery in those patients in which R0 may be obtained with a vascular resection and reconstruction, as coherent with our previous findings [19, 20].

In conclusion, LR and OLT for locally advanced pCCA had similar rates of major complications and post‐operative mortality, but NACR was associated with increased vascular complications. Survival was difficult to compare in the groups due to their heterogeneity, but OLT, especially with NACR, seems to give better results than LR.

## Author Contributions

E.P., F.J.H.H. and B.G.K. were responsible for conceptualization of the paper, analysis and first draft of the paper. E.P., P.B.O., B.G.K. and A.R. coordinated data collection for the resection group. R.J.P., R.A. and F.J.H.H. coordinated data collection for liver transplantation. All authors participated in writing and revising the final version of the paper.

## Funding

The authors have nothing to report.

## Ethics Statement

According to the Central Committee on Research Involving Human Subjects, this type of study, using de‐identified data, does not require approval from an ethics committee.

## Conflicts of Interest

The authors declare no conflicts of interest.

## Supporting information


**Table S1:** Logistic Regression analysis for 90‐day mortality.

## Data Availability

Data were obtained from the Perihilar Cholangiocarcinoma Collaboration Group and from a collaboration between three Dutch and five French transplant centres. The data are available from the corresponding author upon request.
