# Supplementary material for: Outcomes of Liver Transplant Versus Partial Hepatectomy for Perihilar Cholangiocarcinoma Patients Requiring Arterial Reconstruction
Source: Liver Int. 2026 Jun 21;46(7):e70729. doi: 10.1111/liv.70729 (PMC13284446; doi:10.1111/liv.70729)
Supplement: Supplementary file 1 — Table S1: Logistic Regression analysis for 90‐day mortality. [file LIV-46-0-s001.docx]

Table S1. Logistic Regression analysis for 90-day mortality.

| **Variable** | **Univariate** | | **Multivariable** | |
| --- | --- | --- | --- | --- |
|  | **OR (CI 95%)** | **P value** | **OR (CI 95%)** | **P value** |
| **Age** | 1.07 (1.01-1.12) | 0.029 | 1.08 (1.01-1.17) | 0.027 |
| **Sex, male** | 1.15 (0.33-3.98) | 0.82 | - | - |
| **BMI** | 1.18 (1.02-1.37) | 0.029 | 1.16 (0.96-1.40) | 0.12 |
| **CA 19-9 (UI/mL)** | 1.00 (0.99-1.01) | 0.96 | - | - |
| **PSC** | 0.45 (0.05-3.68) | 0.45 | - | - |
| **Preop. Biliary drainage** | 0.17 (0.04-0.61) | 0.007 | 0.15 (0.03-0.73) | 0.019 |
| **Preoperative Cholangitis** | 0.73 (0.16-3.24) | 0.67 | - | - |
| **Bismuth Type IV** | 0.46 (0.14-1.47) | 0.19 | 0.67 (0.14-3.28) | 0.62 |
| **NACR** | 1.25 (0.36-4.37) | 0.72 | - | - |
| **OLT (vs LR)** | 0.64 (0.20-2.06) | 0.46 | - | - |
| **Vascular complications** | 7.2 (2.16-23.99) | 0.001 | 9.60 (2.16-42.60) | 0.003 |

Abbreviations: BMI: body mass index; LR: liver resection; NACR: neo adjuvant chemoradiotherapy; OLT: orthotopic liver transplantation; PSC: primary sclerosing cholangitis.
